# A qualitative study of age-specific care needs in patients with early-onset advanced colorectal cancer

**DOI:** 10.1016/j.pecinn.2026.100474

**Published:** 2026-03-18

**Authors:** Anne Marije Luik, Jacqueline M. Tromp, Tineke E. Buffart

**Affiliations:** Department of medical oncology, Cancer Center Amsterdam, Amsterdam UMC, the Netherlands

**Keywords:** Qualitative research, AYA questionnaire, Age-specific care needs, Early-onset, Advanced colorectal cancer

## Abstract

**Objective:**

To investigate whether a questionnaire for Adolescent and Young Adults (AYAs), used to make an inventory of clinical care needs, can also be helpful to identify care needs of patients with early-onset colorectal cancer (eoCRC) (aged up to 50 years).

**Methods:**

To evaluate the content and usability of the questionnaire, semi-structured interviews with eoCRC patients treated with systemic therapy were conducted until data saturation was reached. Characteristics of respondents were reported. Interviews were transcribed verbatim, and analyses were performed using grounded theory.

**Results:**

EoCRC patients perceived the questionnaire as a user-friendly tool to identify care needs. They long for more support on the themes family, support of children, interaction with family and friends and proactive care planning, depending on their phase of life and disease. Patients desire to discuss the questionnaire multiple times, because care needs change during the disease course.

**Conclusion:**

The questionnaire is a user-friendly tool to identify care needs of eoCRC patients. To improve care, patients emphasize that the questionnaire should be specified by phase of life and disease, include extra topics and offered several times.

**Innovation:**

This study identifies unique care needs of patients with eoCRC and addresses topics to be improved in current care.

## Highlights

• The AYA-questionnaire is a good tool to identify care needs.
• Important to discuss: fertility, sexuality and support of children and family.
• Proactive care planning needs attention.
• The questionnaire should be discussed several times.

## 1 Introduction

The number of patients with early-onset colorectal cancer (eoCRC), defined as patients diagnosed with CRC below the age of 50 years [1], is rapidly increasing with 1–2% annually since the mid-1990's [2], [3]. Models predict that in 2030, 10% and 25% of all patients who are diagnosed with colon and rectal carcinoma respectively, are below 50 years of age [4]. This trend has been observed in several, mainly Western, countries [5].

Patients with eoCRC have different care needs compared to those with average or late- onset CRC, as they are at a different stage in their lives. They face unique problems including fertility, sexual function, body image, career aspirations, financial security and the impact on their young children, all of which negatively affect their quality of life [6].

Additionally, patients with eoCRC report higher levels of fatigue, pain and diarrhea alongside reduced physical, emotional, and social functioning, highlighting their increased need for supportive care [7].

Currently, care for eoCRC patients, with their unique age-specific needs, is generally not well integrated into standard clinical care.

In the Netherlands, age-specific care for Adolescent and Young Adults (AYA), defined as patients with cancer between 18 and 39 years of age, is initiated by the national AYA “Young & Cancer” Care Network [8]. They developed a questionnaire for these patients to make an inventory of age-specific care needs, referred to as the AYA-questionnaire. The questionnaire covers several aspects of daily life that can be affected by a cancer diagnosis. It is unclear whether this AYA-questionnaire can also be used to identify age-specific care needs of eoCRC patients below 50 years of age. Therefore, the aim of this study was to investigate the applicability of the AYA-questionnaire in eoCRC patients with advanced disease, to identify care needs and ultimately improve patient care.

## 2 Methods

### 2.1 Study design and participants

This qualitative study was performed between January 1 to April 1, 2023. Patients diagnosed with advanced or metastatic CRC below 50 years of age, who started systemic therapy within 6 months, were asked informed consent for study participation. Patients were excluded when they were unable to speak and write the Dutch language, or when they were legally incompetent. Eligible patients were identified weekly by the clinical team using the outpatient clinic lists. After obtaining informed consent, the AYA-questionnaire was provided to the patient.

The topics of the AYA-questionnaire are shown in Table 1. The complete AYA-questionnaire is shown in Supplementary Fig. 1. Using the AYA-questionnaire, an inventory was made of age-specific care needs that were relevant at that time for the patient. Support was organized for these actual care needs. After providing the AYA-questionnaire, data collection was carried out through semi-structured interviews within two to three weeks, using a fixed interview schedule to ensure all interviews were conducted to the same standard. The interview schedule is shown in Supplementary Fig. 2. The interviews were audio-recorded and transcribed verbatim. During this interview, the patients' experiences with the content and user-friendliness of the AYA-questionnaire were evaluated. Interviews continued until data saturation was reached, defined as the point at which no new topics were raised during the interviews. The estimated number of patients was 10–12 based on file research at the Amsterdam UMC.

**Table 1:** Topics AYA-questionnaire.

Topics
General (family situation, involved care providers)
Fertility and pregnancy
Illness and treatments
Educations
Work
Eating and drinking
Physical appearance
Sport and exercise
Emotions
Meaning and purpose
Intimacy and sexuality
Friends
Family
Benefits and allowances
Mortgages and insurance
Late effects
Complementary care
Palliative care
Death

The study was approved by the Medical Ethical Review Committee of the Amsterdam UMC (METC-number 2022.0732) and the ethical principles of the Declaration of Helsinki were followed.

### 2.2 Data analysis

Transcripts were systematically collected and analyzed using grounded theory with the steps of Baarda et al. [9]. In the first phase, open coding was done: all text fragments interesting for achieving the objective of the study were identified. The second phase involved axial coding. The codes that had arisen during the first phase were grouped into themes. In the third phase, selective coding, an attempt was made to construct a conceptual model from the axial codes. Relationships and connections were made between the codes in order to answer the objective of the study for the various respondents. To increase the internal validity of the study, peer debriefing was used in which the analyzed and anonymized transcript of the first interview was presented to a fellow nurse practitioner for assessment. The feedback was discussed until consensus.

## 3 Results

In total, ten patients were included in the study. All ten patients completed the AYA-questionnaire followed by a consultation to discuss the age-specific care needs described in the questionnaire. One patient was admitted for emergency surgery shortly after discussing the AYA-questionnaire, which prevented participation in the interview. One patient declined the interview because it was too burdensome. Eight patients participated in the extended interview to further investigate the experiences with the AYA-questionnaire. These interviews took place within two to three weeks after the consultation in which age-specific care needs were discussed and lasted, on average, half an hour each. In the seventh interview no new topics were mentioned, which was confirmed in the eighth interview. As a result, we concluded that data saturation had been reached. Characteristics of the eight respondents are summarized in Table 2.

**Table 2:** Characteristics of respondents.

Characteristics	Participants interview (*n* = 8)
Age	
Median (min, max)	44 (27–49)

Gender	
Male, n (%)	5 (62.5)

Marital status	
Single, n (%)	4 (50)
Living together, n (%)	4 (50)

Family composition	
Children, n (%)	3 (37.5)
No children, n (%)	5 (62.5)

Educational attainment	
Secondary vocational education, n (%)	3 (37.5)
Higher professional education, n (%)	5 (62.5)

Stage of the disease	
Locally advanced n, (%)	4 (50)
Metastatic n, (%)	4 (50)

ECOG-performance status⁎	
0–1 n, (%)	7 (90)
2 n, (%)	1 (10)

⁎ Eastern Cooperative Oncology Group Performance Status.

To ensure the anonymity of the respondents, the quotes used in the results section are numbered and can be traced back to the interview transcript of the concerned respondent.

### 3.1 General impression of eoCRC patients regarding the AYA-questionnaire

All respondents were positive about the AYA-questionnaire. They perceived the questionnaire as a clear representation of many important topics that need to be addressed after the diagnosis of advanced CRC. The questionnaire helped to identify important topics that patients often forgot to address shortly after a diagnosis of advanced cancer.

The respondents mentioned that they preferred to receive the questionnaire before starting systemic treatment, rather than during the treatment (example in quote number 1, Table 3).

**Table 3:** Examples of respondents' quotes on their general impression of the AYA-questionnaire.

Quote number	Quote
1	*“In the beginning I was actually surprised by the message regarding the disease and in my experience, I got on a roller coaster that raced on. I did not have time to really think about all those topics, so the questionnaire is useful to receive. In that way I was kind of forced to think about things that I need to arrange. It gives something to hold on to or some control in a situation I do not control.” (respondent 4, age 48)*
2	*“I would like to discuss the questionnaire a few times. Simply because the situation can change, and new questions or new care needs can arise.” (respondent 4, age 48)*
3	*“The whole part of palliative care and death is of course quite confrontational if you are not actually faced with it yet. It does make me aware of the fact that I am very ill, even when things are going very well at this moment. This topic is part of it, of course, and it got me thinking again when I filled in the questionnaire. I cannot deny the subject. There are many things that I still must arrange for myself but keep postponing… the funeral and the funeral insurance, the inheritance, insurance policies on my name…” (respondent 6, age 44)*

A number of respondents expressed a desire to discuss the AYA-questionnaire multiple times throughout the treatment process, as their medical situation evolved, leading to new or different care needs (example in quote number 2, Table 3).

Some respondents found the AYA-questionnaire confrontational, particularly regarding the topics palliative care and discussing death. These respondents felt physically well at the time of the interview, even though they were receiving treatment in a palliative setting (example in quote number 3, Table 3).

### 3.2 Usability of the AYA-questionnaire for eoCRC patients

The respondents indicated that the text in the AYA-questionnaire was easy to understand, and the tone of the questionnaire was perceived as pleasant. The layout was perceived as good: the respondents liked the short chapters and the schematic structure of the questionnaire. One respondent preferred more space to write at the open-ended questions. According to several respondents, the AYA-questionnaire could be made more compact by combining certain themes, making the questionnaire more clear and preventing the repetition of similar topics across multiple sections (see quote number 1, Table 4). Some respondents would prefer the AYA-questionnaire to be more informative, providing guidance on how to address the various themes discussed (see quote number 2 and 3, Table 4).

**Table 4:** Examples of quotes of the respondents on the usability of the AYA-questionnaire.

Quote number	Quote
1	*“Education and work, for example, were now two separate themes. In my opinion they can be merged. I think the theme of emotions and meaningfulness are overlapping, that could also be merged. Benefits and reimbursements, and mortgages and insurance can be merged to the theme financial.” (respondent 1, age 43)*
2	*“I would rather immediately receive an information folder about nutrition, sports and exercise, so that I can immediately read how I can approach this” (respondent 7, age 47)*
3	*“Maybe a checklist? With what should be paid attention to in the event of your death, for example arranging your car insurance and your joint bank account” (respondent 8, age 49)*

### 3.3 Additional topics for eoCRC patients in the AYA-questionnaire

Opinions about the content of the AYA-questionnaire varied. While no respondents wanted to remove any themes from the questionnaire, there was a range of suggestions for additional topics that respondents would like to see included or explored in more detail. For example, the respondents with children would like more attention to be given to the theme family and support of the children. This theme could be discussed in more detail, including the different age categories of children, the problems they may experience, and how patients can address these challenges (see quote number 1, Table 5). A theme that several respondents felt was missing from the questionnaire is how to interact with family and friends. The respondents experienced a lot of support from those around them, but they found it difficult to express their limits when the support became too much (see quote number 2, Table 5).

**Table 5:** Examples of quotes of the respondents on additional topics in the AYA-questionnaire.

Quote number	Quote
1	*“A theme that I missed in the questionnaire was the theme family because that is the most important thing for me. Occasionally there were questions that refer to the situation at home, but specifically I think a specific theme of family would be useful. And then maybe add ‘child questions’ classified by phase: babies, school-going children, teenagers…. And then ask whether the practical things are still possible: bringing and picking up from swim*min*g lessons, sports clubs, children's parties… And of course, also the emotions of the children and what you can do to support that” (respondent 1, age 43)*
2	*“I do have a few in-depth questions about the theme friends. Am I able to say no when it is too much? How do I deal with the overwhelming support that the people around me want to give me? Which support is helpful, and which is not? And can I also discuss this? What do I allow and what not? I just said to friends: you can do this and nothing more. Also, with apps for example. I do not feel the need to constantly respond to that, but only when I feel like it and feel good enough, I will respond. In the beginning I felt a bit guilty when I did not respond, because people like to do something, but now I do it easier.” (respondent 5, age 27)*
3	*“What is quality of life for me? And how long would I like to continue treatments? Would I consider euthanasia or not when I have exhausted all treatment options? Those are things that keep me busy.” (respondent 5, age 27)*
4	*“Maybe it should include something about what you can do to make it a little more relaxed for yourself, so what do you need to feel a little better and get a little distraction? For me that was, for example, taking a bath or meditating or doing a yoga class. Include a theme of relaxation perhaps? What can you do besides surrender to all the treatments and all the medicines to feel good?” (respondent 3, age 34)*

A couple of respondents lacked the theme proactive care planning, and the themes palliative care and talking about death in the questionnaire, which concerns a conversation about treatment wishes and limits, and end-of-life issues (see quote number 3, Table 5). Several respondents considered the theme relaxation as an important topic. According to them, this theme was given too little attention in the questionnaire (see quote number 4, Table 5).

### 3.4 The opinion of eoCRC patients on the use of the AYA-questionnaire

The respondents indicated that the AYA-questionnaire can be a useful tool to guide discussions about age-specific care needs. They were satisfied with the conversation about their current age-specific care needs based on the questionnaire (see quote number 1, Table 6). One respondent mentioned that it was difficult to discuss the theme sexuality. The patient was not in a relationship and got the impression that care providers considered information about this theme less important for single patients (see quote number 2, Table 6). Aside from this, respondents indicated that no other topics were difficult to discuss. All respondents with children indicated that the topic children and family was the most important topic for them to discuss with the healthcare provider. The support of the children in the parents' disease process was indicated as the most important topic. The respondents without children considered fertility and sexuality as the most important themes to discuss, as well as the interaction with family and friends (see quote number 3, Table 6).

**Table 6:** Examples of quotes of the respondents on the conversation using the AYA-questionnaire.

Quote number	Quote
1	*“That conversation was nice. It is an accessible way to indicate that I am experiencing problems with certain topics without being immediately forwarded for professional help. It is nice to have a moment to discuss such a questionnaire for myself. Also: am I really not experiencing any problems? I already have so many things to think about!” (respondent 1, age 43)*
2	*“Well, I am not really a prude person, but I found it difficult to talk about sexuality because I do not have a partner. I am still young, and I might go on a date with someone. How does that work? If I kiss someone or have sex with someone, am I making them sick because I am being treated with chemotherapy?” (respondent 3, age 34)*
3	*“Well, I no longer have any desire to have children, but how do I know that I have really become infertile due to the chemotherapy? Or does that come back when I do not get chemotherapy anymore? Do I always have to keep having sex with my wife with a condom or can I stop? Those are things I think are important to discuss.” (respondent 7, age 47)*

## 4 Discussion and conclusion

### 4.1 Discussion

In this qualitative study the experience with the AYA-questionnaire was investigated in patients below 50 years with advanced CRC. This study shows that all patients are positive about the questionnaire and indicated that the questionnaire can be a useful tool to identify age-specific care needs, not only for patients between 18 and 39 years but also for patients between 40 and 50 years. Despite positive experiences with the questionnaire, patients with eoCRC lacked further in-depth inventories on several topics such as family and support of the children, how to interact with family and friends, and proactive care planning. Additionally, patients expressed a preference for receiving the questionnaire before the start of treatment and for discussing the topics several times throughout the treatment process and disease course, as care needs can change. The frequency of discussing the questionnaire during the treatment process has not been defined. Also, patients did not specify whether they preferred to discuss the questionnaire with the nurse practitioner or the oncologist. Both the frequency and the discussing partner are probably different for each individual patient.

These study findings are crucial since the incidence of eoCRC is increasing rapidly and, in the near future, a substantial part of patients with CRC will be below 50 years. This study, as well as previous studies, [6], [7] shows that young patients with CRC have different care needs compared to older patients, which can negatively impact their quality of life. For AYA patients, defined as patients between 18 and 39 years, inventories of care needs and support have been integrated in clinical care in multiple hospitals. However, this overlooks the 40–50 age group who, as shown in this study, face similar problems and have comparable care needs,.

A recent cross-sectional questionnaire study on age-specific care needs in young patients with cancer below 40 years showed that the AYA-questionnaire provides a good reflection of these needs [10]. This is partly because the AYA-anamnesis from the AYA care network was used in this study to identify age-specific care needs [8]. The AYA-questionnaire used in the present study was based on this similar AYA-anamnesis. Results from the cross-sectional questionnaire study also indicated that the themes children and family, and interaction with family and friends were not sufficiently addressed, in agreement with the present study.

Palliative care and discussing death were mentioned as underexposed topics in our study, which has not been described in the previous mentioned cross-sectional questionnaire study. This can be explained by the fact that the cross-sectional questionnaire study was conducted with young cancer survivors, in contrast to the majority of the patients in the present study, who were more directly confronted with end-of-life issues. The importance of palliative care and the associated proactive care planning for young patients with cancer has been demonstrated previously, highlighting that patients below 40 years do want to discuss end-of-life matters, including their wishes and needs. This is consistent with the results of the current study [11].

Fertility and sexuality are additional important topics to discuss with patients, as shown in the present study. Previous qualitative research using focus groups also showed that patients consider it very important to discuss the impact of treatment on sexuality. However, they reported difficulties in addressing this topic with their care providers [12]. In the present study only one patient considered it difficult to discuss sexuality. A possible explanation could be that the average age of the patients in the previous study is sixty-three, and older patients may find it more difficult to discuss sexuality than younger patients. Another explanation could be that in our study sexuality was discussed by the nurse practitioner. It has been reported that patients find nurse practitioners very accessible and feel comfortable to discuss various topics with them, including sexuality. Patients rather discuss medical issues with their treating oncologist and additional topics with the nurse practitioner [13]. Currently, the FROSA study is ongoing in the Netherlands to study the influence of cancer on fertility and sexuality in patients who received cancer treatment. Results have to be awaited; however, they could also be important for eoCRC patients receiving palliative treatment as the present study highlights fertility and sexuality as key clinical needs.

Importantly, the results of the present study emphasize that age-specific care needs rather depend on the phase of life and the phase of disease of the patients and not just by the age itself. As demonstrated in our study as well as in a previous study, patients with children have other care needs than patients without children [14]. Patients with cancer between 30 and 40 years are often in a different phase of life, leading to different care needs compared to those under 30 years. They may have more similar needs to patients in the 40–50 age group than those in the under-30 group. Additionally, the trend towards delayed childbearing leads to shifts in clinical needs, highlighting that phases of life must be considered when assessing care needs.

This study has several limitations. First of all, this study is based on interviews on a small number of patients. However, data saturation was reached, indicating that increasing the number of interviews with patients treated at our hospital is unlikely to provide additional insights. Second, this study was conducted at a single center in the Netherlands, which may limit the generalizability of our conclusions to a broader European or non-European eoCRC population. However, the topics covered in the questionnaire could serve as a foundation for discussing care needs across patients from different countries and cultures. The identified gaps in care may vary, and further exploration in other countries could be valuable to address all individual care needs, especially considering differences in access to medical treatment and availability of psychosocial support. These contextual variations may shape patient and caregiver experiences, which could limit the generalizability of our study findings.

Third, all patients had already started their systemic treatment before discussing the AYA-questionnaire. While patients expressed a preference for receiving the questionnaire before starting treatment and multiple times during the disease course, the additional value of administering the questionnaire prior to treatment could not be confirmed in the present study.

Fourth, most patients were in a good clinical condition with an ECOG performance status of ≤1, suggesting the potential of selection bias [15]. Patients with a poor performance status may have different care needs than those represented in this study. Their care needs may include more comprehensive symptom management (e.g., pain relief, fatigue), assistance with daily living activities, and increased psychosocial support, which could influence the outcomes of this study. On the other hand, generally most young patients have a good performance status, making the representability of the study results highly likely.

Finally, only Dutch-speaking patients with a Western background were included, while patients with a non-Western background may have different age-specific care needs that should be considered in discussing those needs. Previous Dutch studies involving patients with a Turkish or Moroccan background showed that perceptions of good quality care may differ. Whereas Dutch healthcare providers emphasize high-quality palliative care through proactive care planning, Turkish and Moroccan cultural perspectives tend to prioritize curative treatment until the end of life [16]. Therefore, the findings of this study have limited generalizability to the broader population, particularly among patients with a non-Western background.

### 4.2 Innovation

This study highlights the importance of addressing the care needs of patients with eoCRC and identifies key topics requiring attention, such as fertility, sexuality, support for children and family, and proactive care planning. Additionally, eoCRC patients emphasize the need to revisit these topics multiple times throughout the disease course, as care needs may change over time. The results of this study reinforce the necessity of integrating these considerations into routine clinical care, alongside anti-cancer treatment, for every eoCRC patient.

### 4.3 Conclusion

This study shows that eoCRC patients with advanced disease perceive the questionnaire, designed for AYAs aged 18–39, as a useful tool to identify their age-specific care needs. Fertility, sexuality and support of children and family are important topics to address. The themes family and support of children, interaction with family and friends and proactive care planning need increased attention.

The questionnaire can be improved by specifying the questionnaire by phase of life and phase of disease and not just by age. Furthermore, life phases and its associated care needs can change during the treatment process. Therefore, consideration should be given to discuss the questionnaire at several timepoints during the disease course.

## CRediT authorship contribution statement

**Anne Marije Luik:** Writing – original draft, Project administration, Methodology, Investigation, Formal analysis, Data curation, Conceptualization. **Jacqueline M. Tromp:** Writing – review & editing. **Tineke E. Buffart:** Writing – review & editing, Supervision, Methodology, Formal analysis, Conceptualization.

## Funding

This research did not receive any specific grant from funding agencies in the public, commercial, or not-for-profit sectors.

## Declaration of competing interest

The authors declare the following financial interests/personal relationships which may be considered as potential competing interests:

T. Buffart reports a relationship with Pierre Fabre Oncology, Servier, Amgen, Nordic Pharma (Speaking and lecture fees), BMS (Consultation or advisory fees) that includes: consulting or advisory and speaking and lecture fees.

J. Tromp reports a relationship with Amsterdam University Medical Centres that includes: non-financial support.

A.M. Luik reports a relationship with Pierre Fabre Oncology, Servier, Amgen, Nordic Pharma (Speaking and lecture fees) that includes: speaking and lecture fees.

## Appendix A Supplementary data

Supplementary material 1 Supplementary Fig. 1: AYA questionnaire. Supplementary material 1

Supplementary material 2 Supplementary Fig. 2: Interview schedule. Supplementary material 2
