# Supplementary figures and images for: A qualitative study of age-specific care needs in patients with early-onset advanced colorectal cancer
Source: PEC Innov. 2026 Mar 18;8:100474. doi: 10.1016/j.pecinn.2026.100474 (PMC13022696; doi:10.1016/j.pecinn.2026.100474)

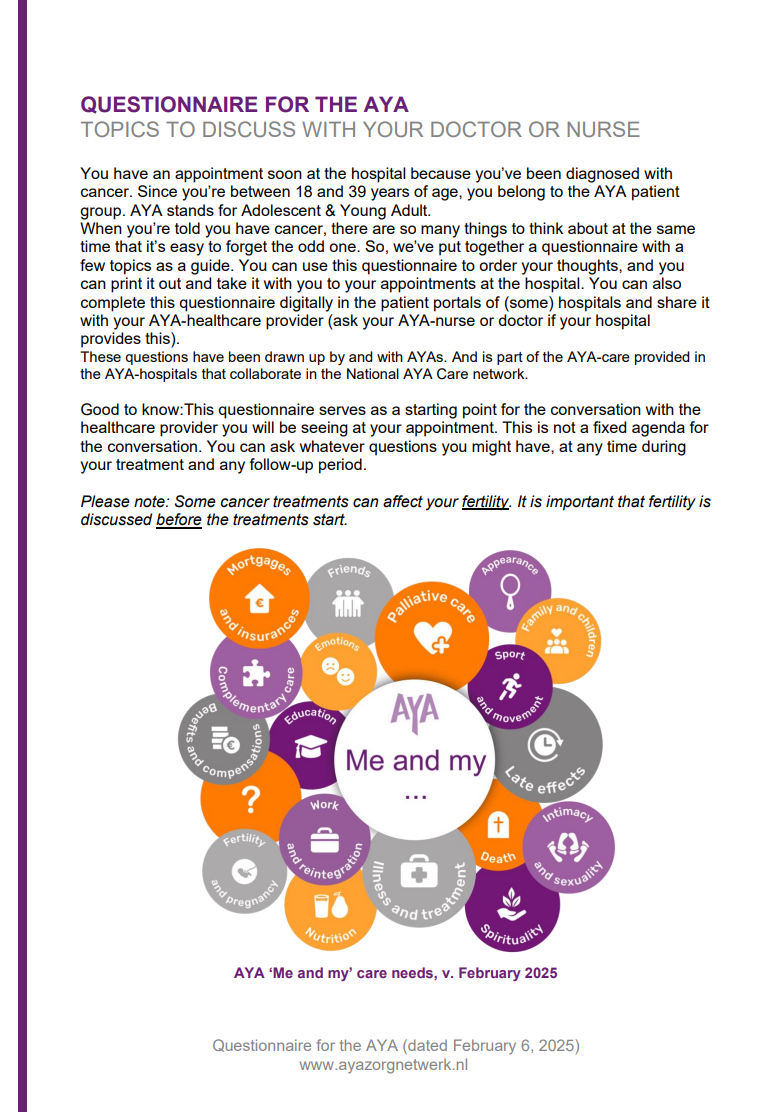


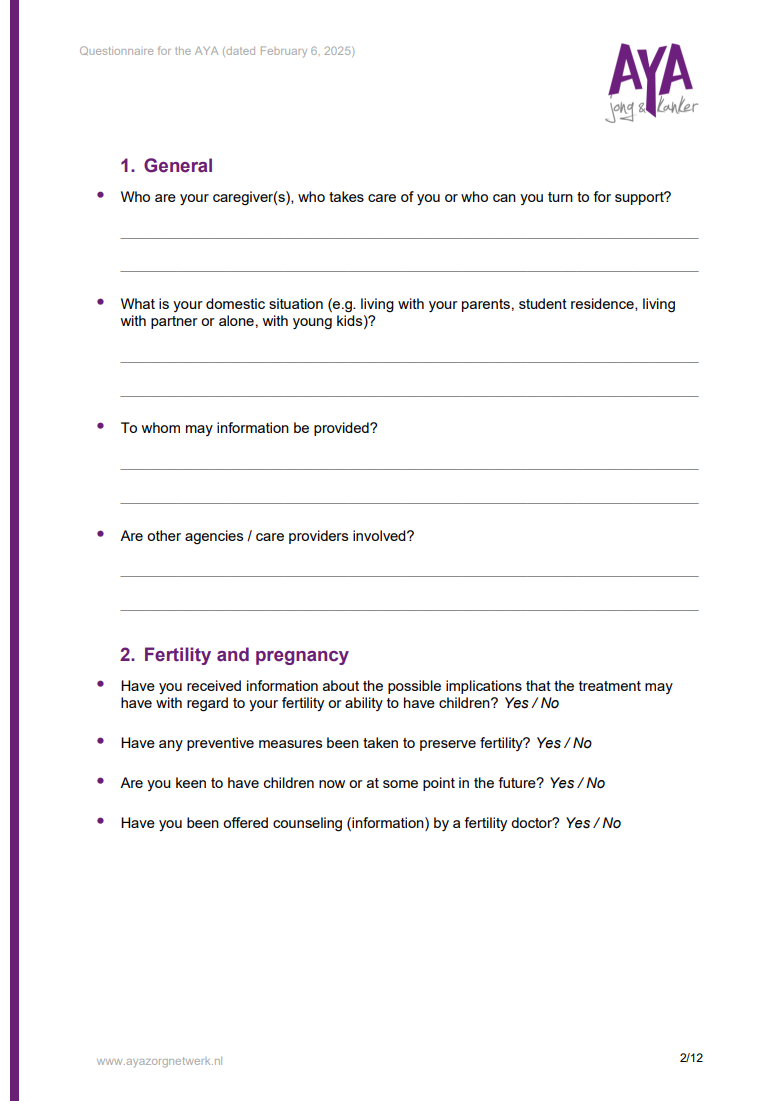


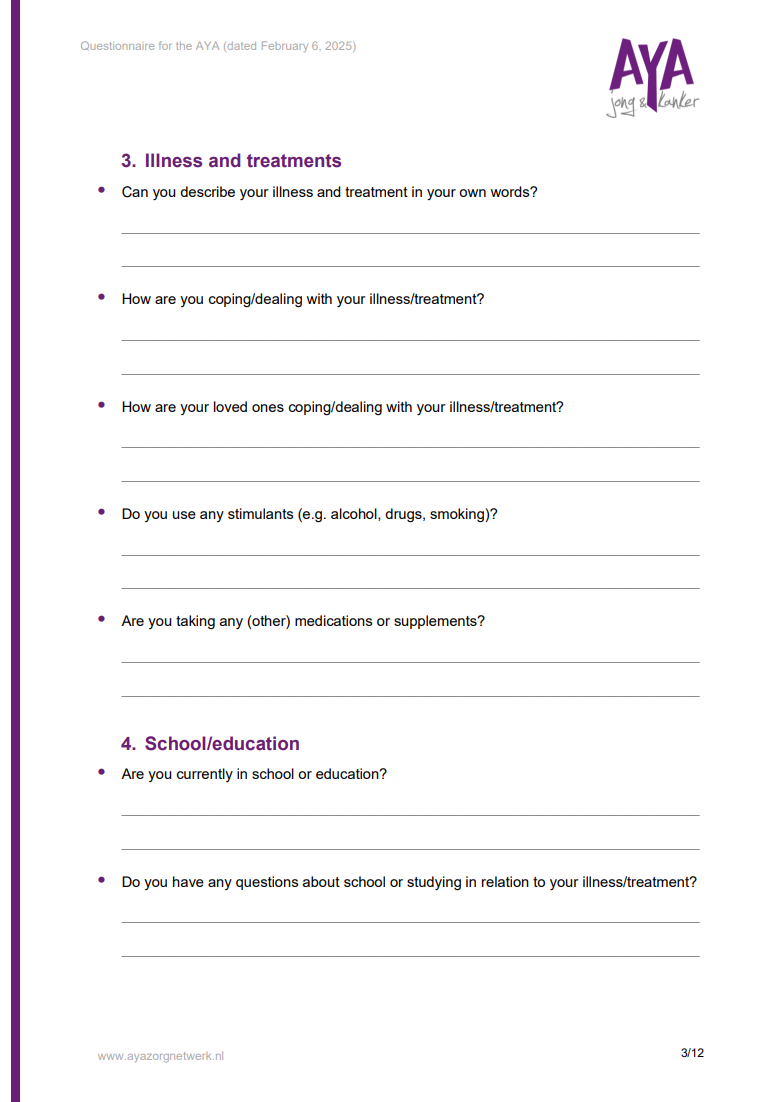


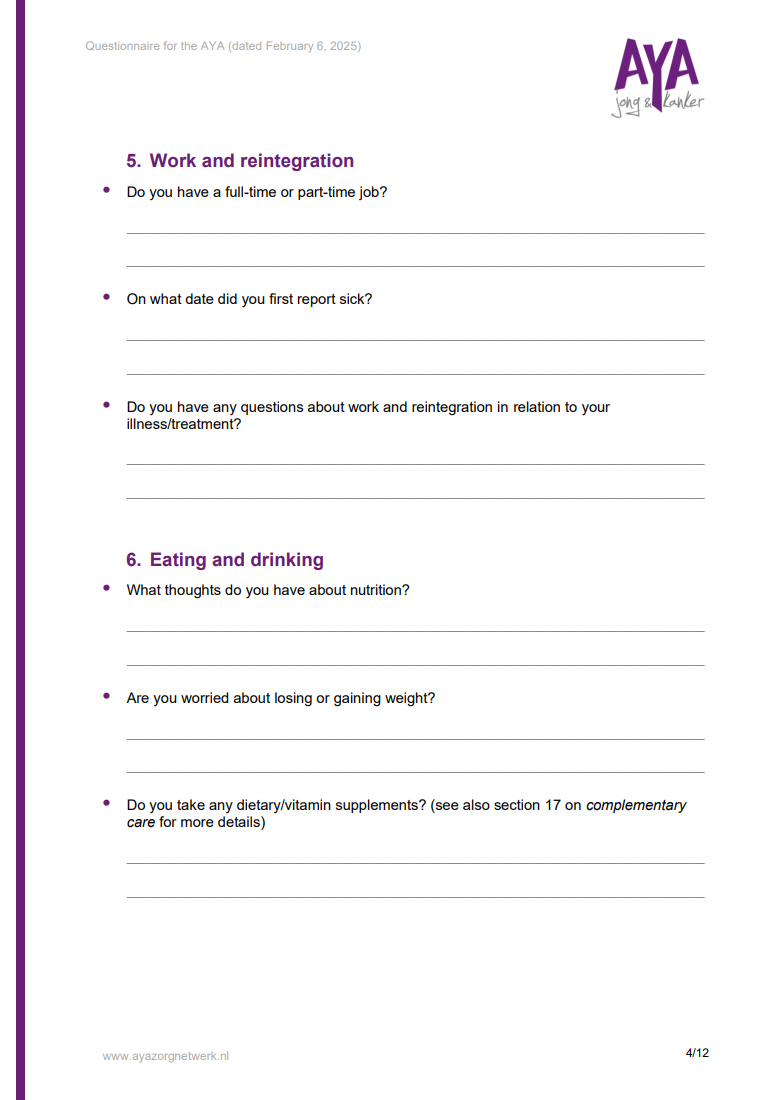


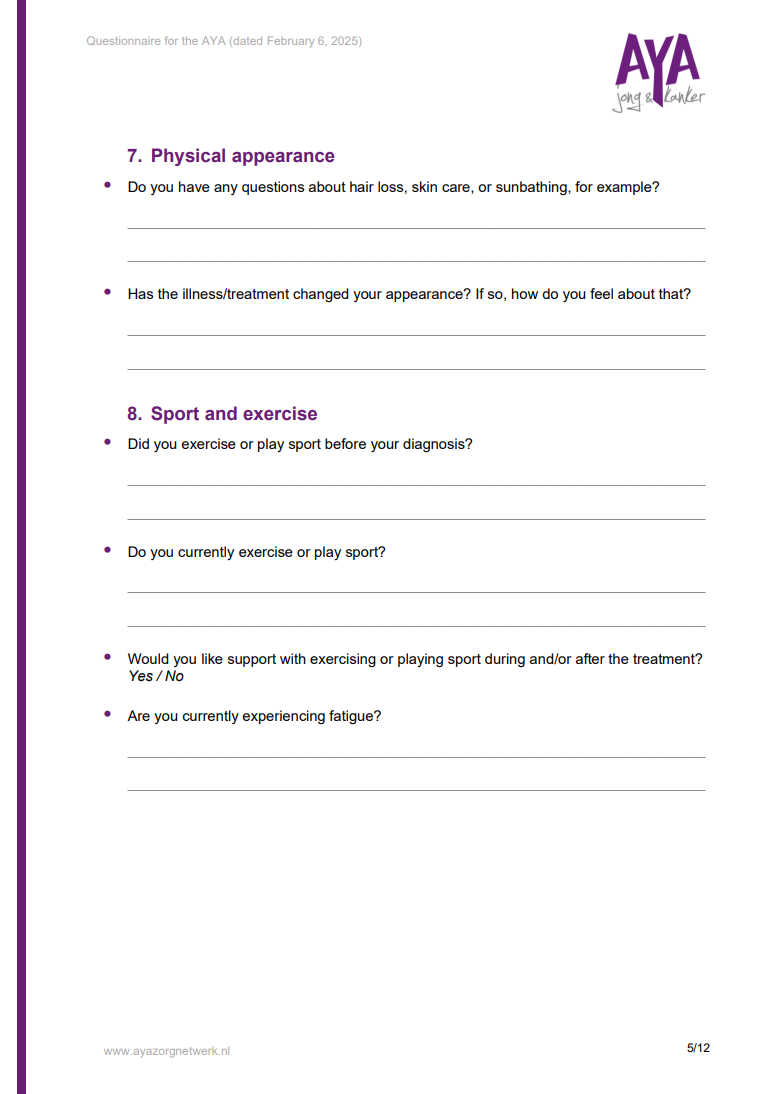


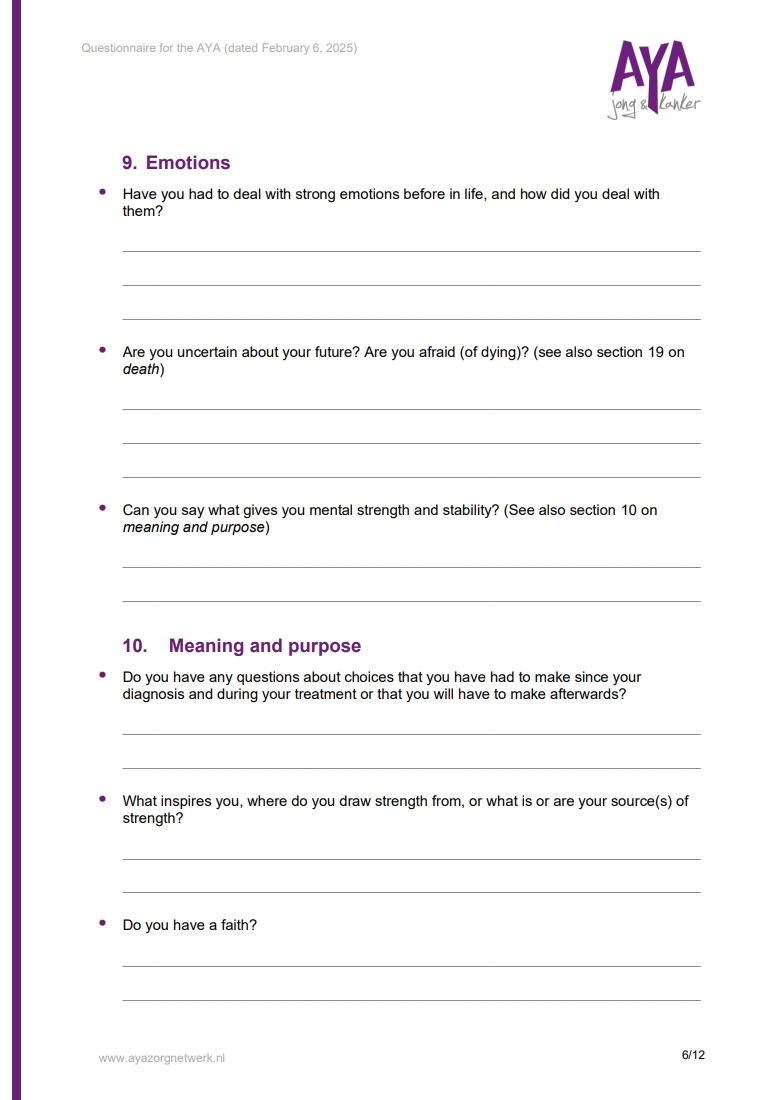


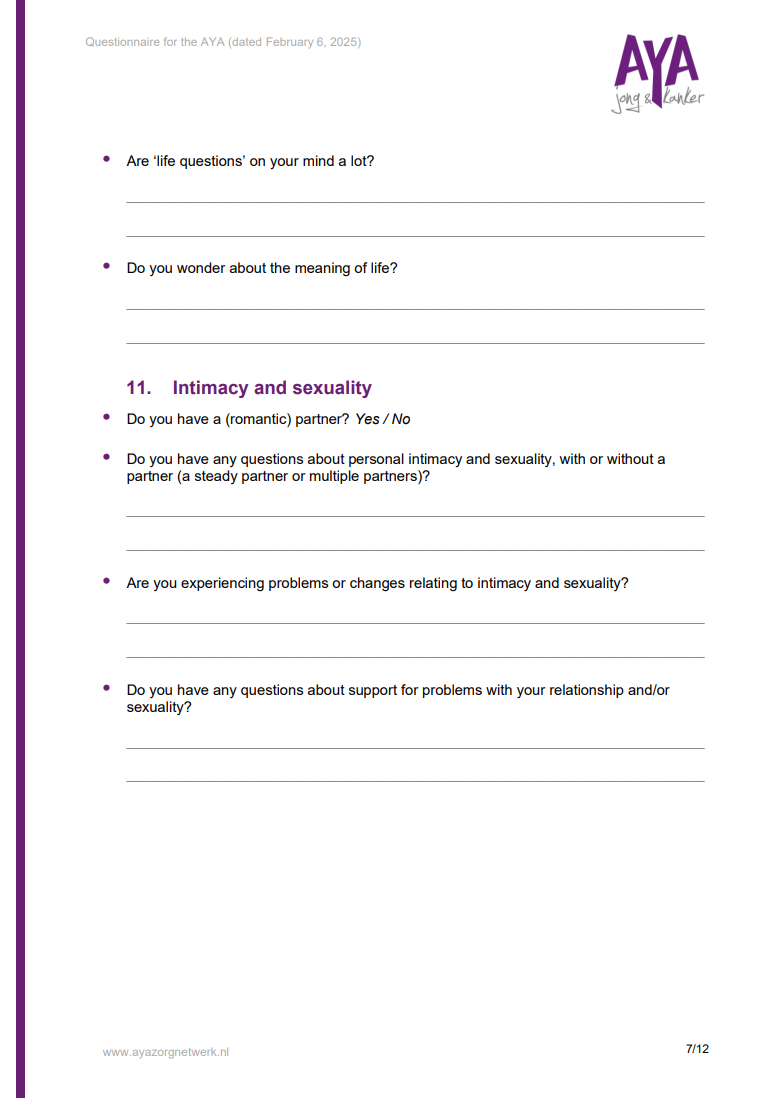


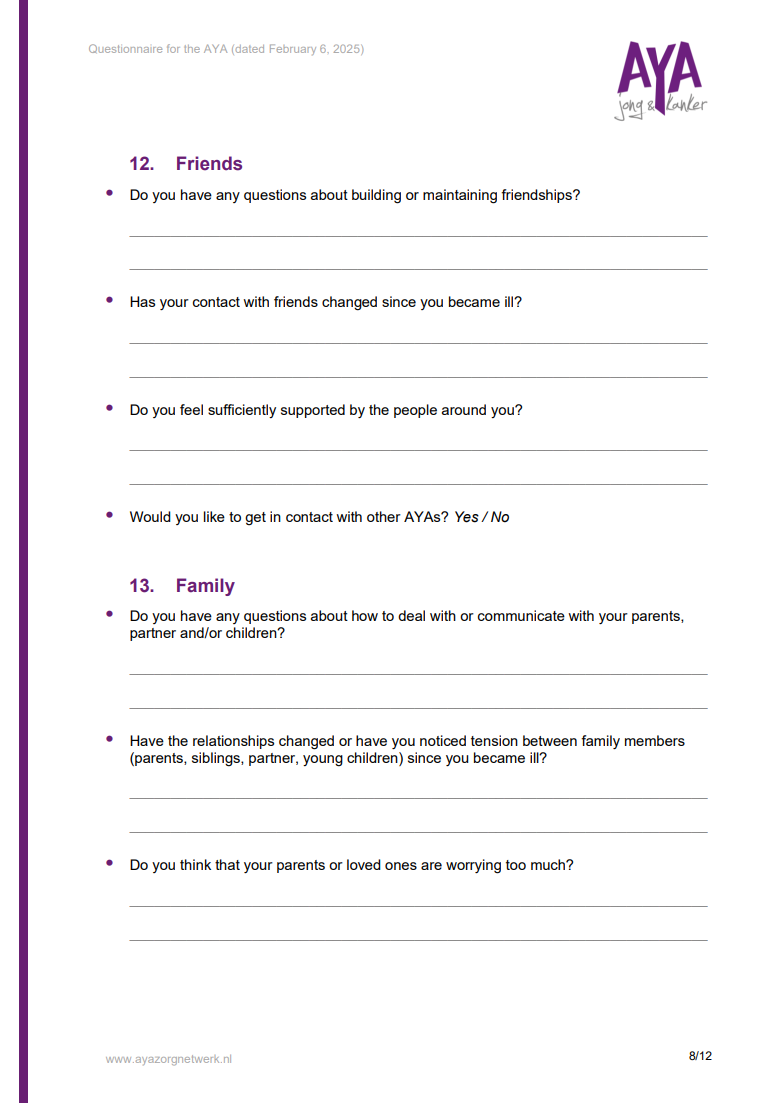


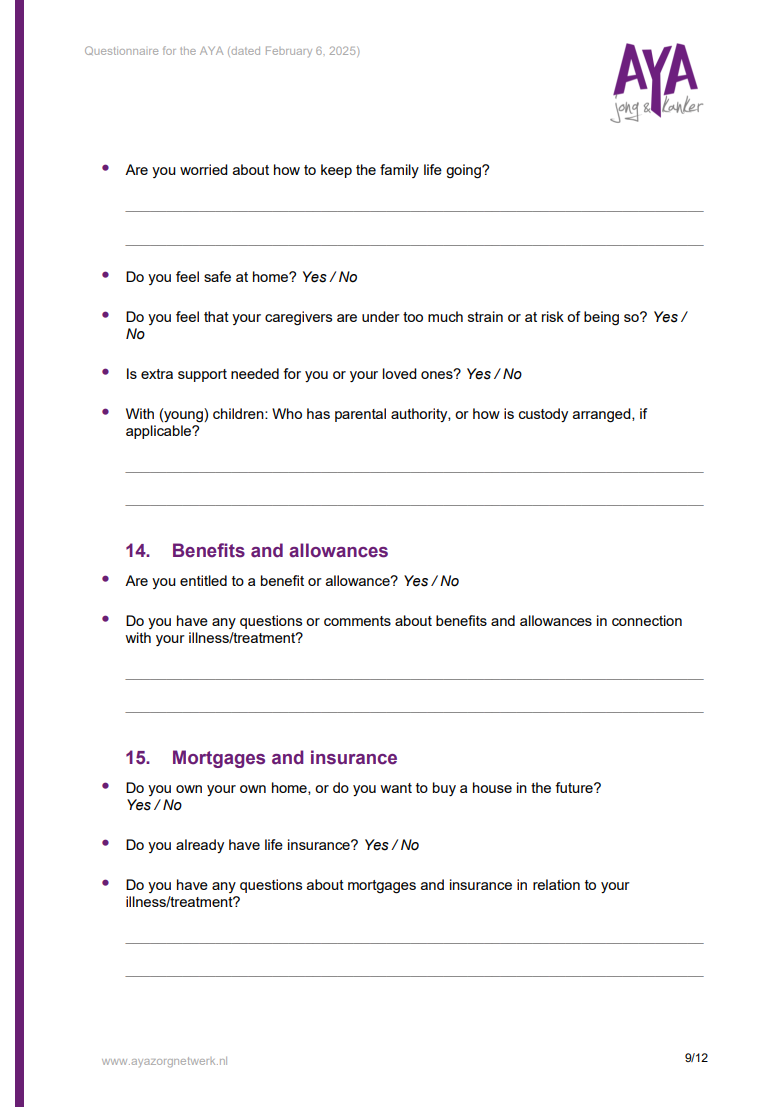


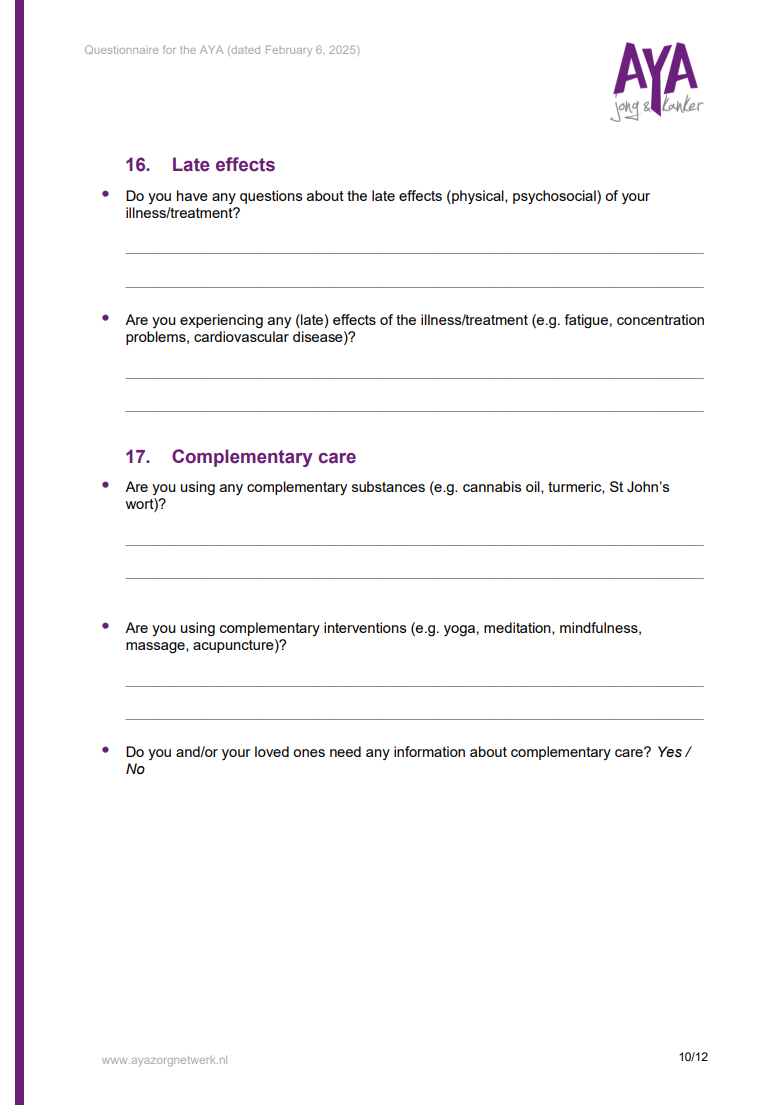


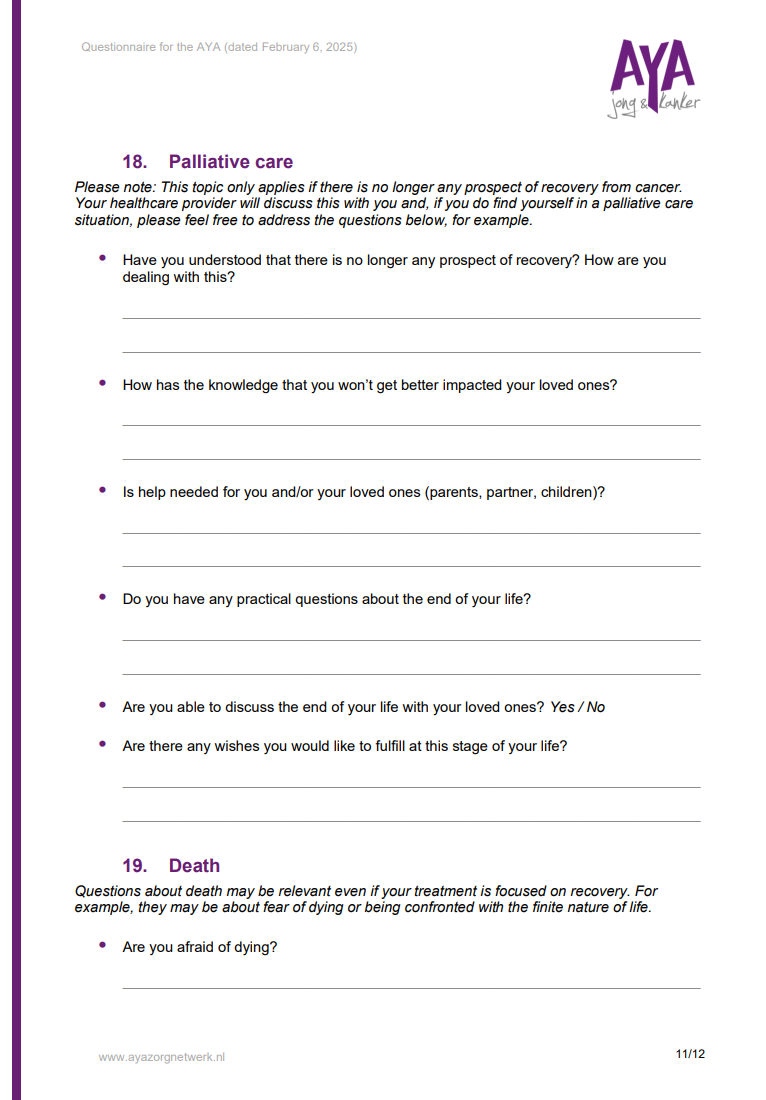


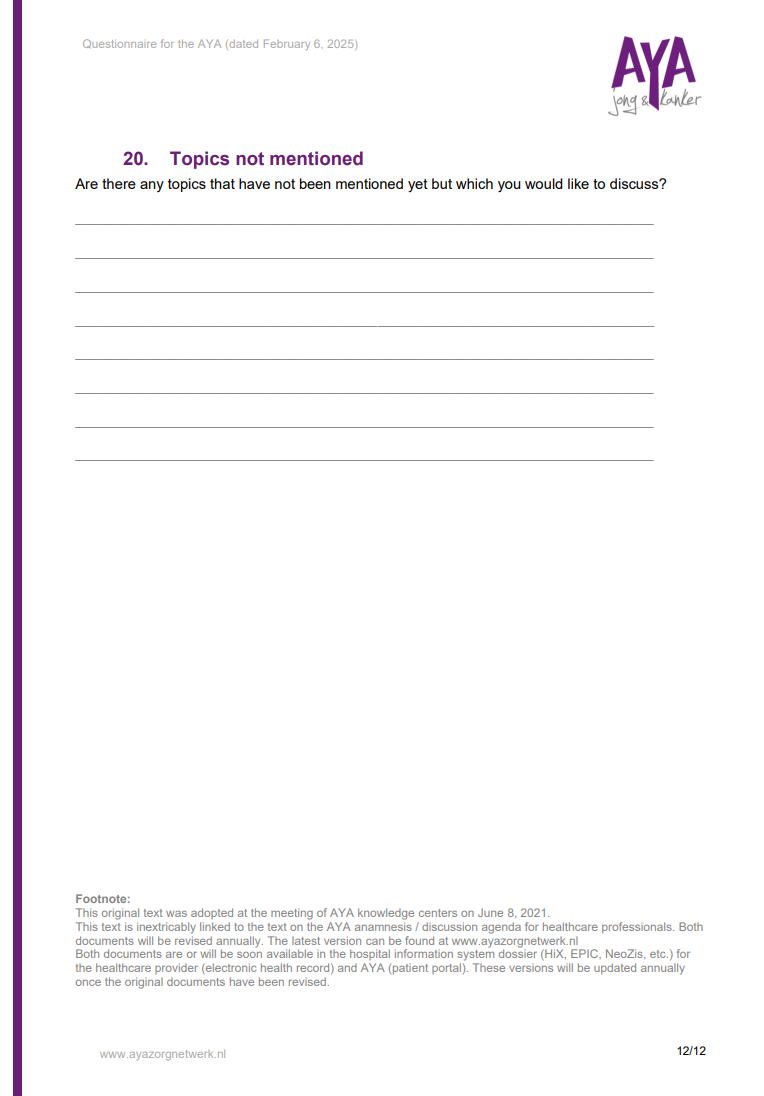

Supplement: Supplementary material 1 — Supplementary Fig. 1: AYA questionnaire. [file mmc1.docx]
