# Supplementary material for: A qualitative study of age-specific care needs in patients with early-onset advanced colorectal cancer
Source: PEC Innov. 2026 Mar 18;8:100474. doi: 10.1016/j.pecinn.2026.100474 (PMC13022696; doi:10.1016/j.pecinn.2026.100474)
Supplement: Supplementary material 2 — Supplementary Fig. 2: Interview schedule. [file mmc2.docx]

| **Questions** |
| --- |
| **What did you think of the content of the AYA-questionnaire?**  Sub questions:   - Which topics from the questionnaire do you think are essential to discuss? - Which topics would you like to add to the questionnaire? - Which topics would you like to see formulated differently in the questionnaire? - Which topics would you like to remove from the questionnaire? - Are there underexposed topics that are important to you which are not addressed in the questionnaire? - Which topics were most important for you to discuss? |
| **How user-friendly did you find the AYA-questionnaire?**  Sub questions:   - How easy were the texts in the questionnaire to understand? - What was your experience with the intonation of the questionnaire? - What was your experience with the lay-out of the questionnaire? - What was your experience with the structure of the questionnaire? - What was your experience with the timing of the questionnaire, in your case:   0 Before starting treatment  0 During treatment |
